# Acute cardiovascular responses to slow and deep breathing in normotensive men and women

**DOI:** 10.1113/EP093086

**Published:** 2026-04-09

**Authors:** Malika Felton, Vikram Mohan, Vanora A. Hundley

**Affiliations:** ^1^ School of Allied Health and Exercise Science Bournemouth University Bournemouth UK; ^2^ School of Health and Care Bournemouth University Bournemouth UK

**Keywords:** blood pressure, RESPeRATE, respiratory sinus arrhythmia, slow and deep breathing

## Abstract

Slow and deep breathing is recommended as an effective treatment for hypertension using the RESPeRATE device. However, the acute cardiovascular responses to slow and deep breathing, including the potential mechanisms underlying its antihypertensive effect, are not fully understood. This study characterised the acute cardiovascular responses to three differing, 10‐min bouts of slow and deep breathing. Twelve participants completed four conditions in a randomised order: (1) RESPeRATE, (2) dynamic slow and deep breathing frequency, (3) fixed breathing frequency of 6 breaths min^−1^, and (4) spontaneous breathing. Comparing mean values for all variables obscured the cardiovascular perturbations created by slow and deep breathing. However, intra‐ and inter‐breath differences (minimum vs. maximum) in arterial blood pressure were significantly larger during slow and deep breathing compared with spontaneous breathing. The amplitude of systolic blood pressure oscillations increased by up to 10.2% (11.4 mmHg) during inspiration and 8.4% (10.0 mmHg) during expiration (spontaneous breathing; 2.9% (3.4 mmHg) and 3.4% (4.2 mmHg), respectively). Cardiovascular responses were maximised at ∼6 breaths min^−1^, but further research is needed to identify the optimal breathing frequency to induce maximal cardiovascular perturbations.

## Abstract (highlights)

- **What is the central question of this study?**
  Are there differences in cardiovascular responses to different methods of slow and deep breathing (SDB) delivery, and does utilising a multi‐parametric approach to measuring cardiovascular variables reveal new/different responses?
- **What is the main finding and its importance?**
  The main finding is that all SDB conditions elicited similar cardiovascular responses to each other when compared with spontaneous breathing. However, lower breathing frequencies elicit greater blood pressure oscillations, and higher breathing frequencies (∼8 breaths min^−1^) may not fully optimise cardiovascular responses. This has implications on the practice of SDB for management of hypertension.

## 1 INTRODUCTION

Daily practice of slow and deep breathing (SDB; ≤10 breaths min^−1^) has been recommended by the American Heart Association (AHA) as an effective treatment for hypertension (Brook et al., 2013). Specifically, the AHA recommends the RESPeRATE device, which reduces breathing frequency progressively using auditory tones. The RESPeRATE device has been researched extensively and shown to reduce blood pressure (BP) in hypertensive individuals, when practised daily for 4–8 weeks (Landman et al., 2013; Viskoper et al., 2003). A recent meta‐analysis (Chaddha et al., 2019) found SDB interventions, including RESPeRATE, induced a significant reduction in systolic BP (SBP) and diastolic BP (DBP) of −5.62 mmHg and −2.97 mmHg, respectively.

Despite the apparent health benefits associated with SDB, there is a lack of conclusive information relating to the mechanism(s) underlying its apparent antihypertensive effect (Gerritsen & Band, 2018). Accordingly, these mechanisms are not fully understood and there is a limited understanding of the acute cardiovascular responses to SDB that might underpin its anti‐hypertensive effect.

Recent debate about the appropriate analysis of cardiovascular variability suggests that multi‐parametric approaches to analysing multiple variables are needed to provide a more complete picture of the dynamics of cardiovascular variability (Castiglioni & Parati, 2011). Previous research has taken a singular approach to exploring the cardiovascular responses during SDB. For example, Calcaterra and colleagues investigated the acute effects of SDB upon baroreflex sensitivity and arterial function (pulse wave velocity and augmentation index), but did so in separate studies (Calcaterra et al., 2013, 2014). It is reasonable to assume that breathing‐related fluctuations in variables such as stroke volume and BP are related to any mechanisms that underpin anti‐hypertensive effects of SDB, and therefore studies should measure instantaneous, multi‐parameter haemodynamic responses to SDB to investigate all variables simultaneously.

There is also a discussion about the optimal breathing frequency for SDB delivery, with RESPeRATE using a dynamic breathing frequency that adapts throughout use (aiming to produce a breathing frequency of <10 breaths min^−1^). However, many researchers regard the optimal SDB frequency to be ∼6 breaths min^−1^ (Cullins et al., 2013; Russo et al., 2017). Additionally, as respiratory sinus arrhythmia (RSA) is widely accepted to increase during SDB (Joseph et al., 2005), and as RSA has been suggested to be the main driver of blood pressure variability (Elstad et al., 2001), then there is an argument that using a SDB frequency that maximises RSA could produce maximal changes in other cardiovascular variables in response to the SDB.

The aim of the present study was therefore to characterise the acute cardiovascular responses to SDB using a number of cardiovascular variables and a multi‐parametric approach. Additionally, the physiological responses were compared across different SDB conditions including the AHA recommended device RESPeRATE, a fixed breathing frequency of 6 breaths min^−1^, and a dynamic algorithm that aimed to maximise RSA.

## 2 METHODS

### 2.1 Ethical approval

The experimental protocol was approved by Bournemouth University's Research Ethics Committee (ID 20679) and all experiments conformed to the *Declaration of Helsinki*, except for registration in a database. Written informed consent was obtained from all participants prior to participating in the study.

### 2.2 Participants

Twelve participants took part in the study (6 males and 6 females). All participants were non‐smokers with no current diagnosis of cardiovascular or respiratory disease. No participants were pregnant at the time of taking part. Participants refrained from eating for 2 h and from caffeine, strenuous exercise and alcohol for 12 h prior to data collection.

### 2.3 Slow and deep breathing protocol

Participants completed three controlled breathing conditions and one spontaneous breathing condition in a randomised order. Randomisation was conducted using a random number generator (www.randomizer.org). All breathing conditions were 10 min in duration with a 10‐min period of normal non‐paced breathing prior to each measurement. A 10‐min intervention has been used in previous studies of daily SDB practice using RESPeRATE and was found to be effective at reducing BP (Chaddha et al., 2019). Participants rested at baseline for 5 min prior to starting the protocol, in addition to the 10 min of normal non‐paced breathing prior to the first breathing condition to ensure cardiovascular variables were in a stable, resting state. During the spontaneous breathing condition (S*f*_r_), participants were instructed to breathe normally and no visual feedback was provided to control breathing. The three SDB conditions were (1) RESPeRATE (R*f*_r_), (2) a dynamic algorithm driven by RSA (D*f*_r_), and (3) a fixed breathing frequency of 6 breaths min^−1^ (6F*f*_r_).

The RESPeRATE device (Intercure Ltd., Lod, Israel) gradually lowers breathing frequency as users breathe in time with a fluctuating musical tone. Breathing frequency is reduced to ≤10 breaths min^−1^ and is measured using a belt worn around either the chest or the upper abdomen. A full description of RESPeRATE can be found in Gavish (2010) and Cernes & Zimlichman (2017). Participants completed the dynamic breathing frequency condition (D*f*_r_) using a novel, bespoke biofeedback algorithm that guided breathing dynamically to a personalised frequency. The algorithm created a dynamically driven target breathing frequency, which strived to maximise cardiovascular perturbation, using the amplitude of RSA as the controlled variable. The algorithm used data measured from a finger sensor (photoplethysmography), which tracked the user's instantaneous physiological responses to their breathing. The finger sensor was connected via the headphone socket of an iPad, which ran the software algorithm and provided visual biofeedback to guide breathing frequency.

**FIGURE 1:**
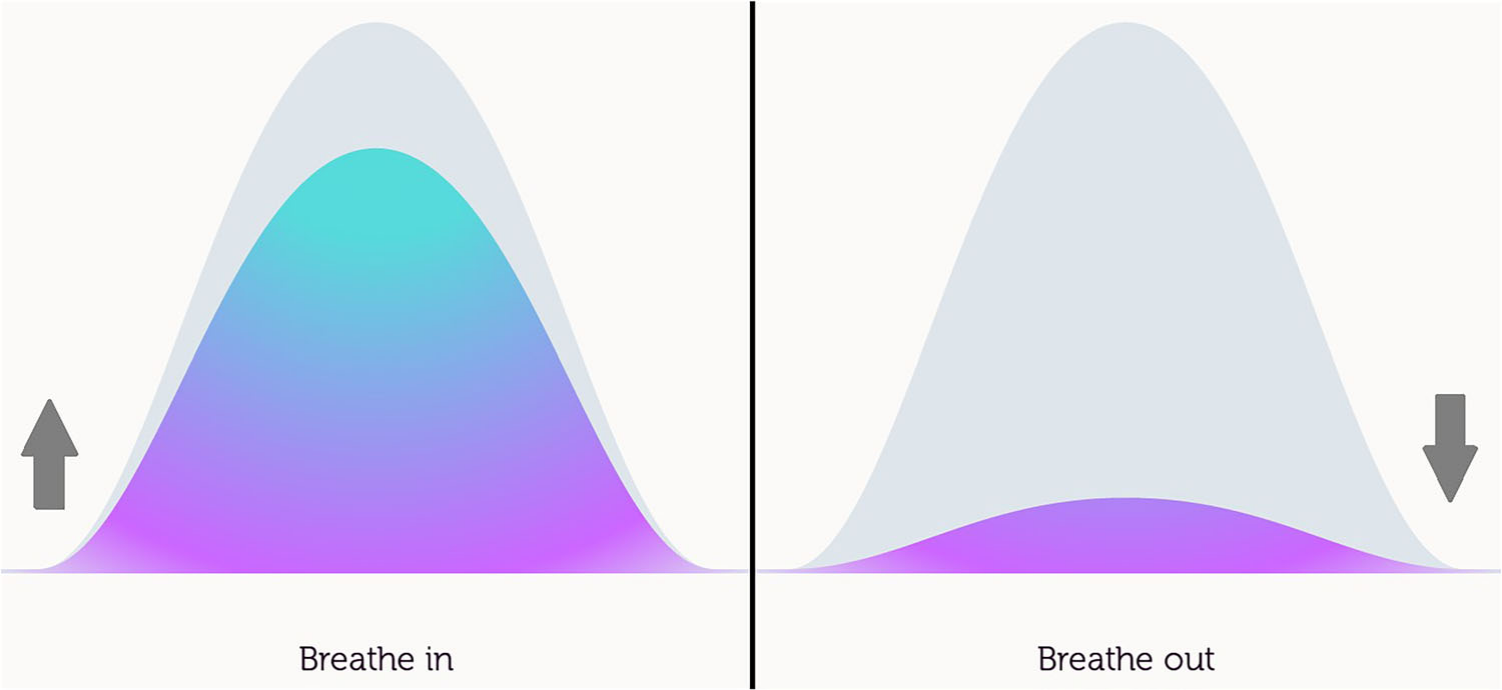
Screenshots of the app graphic. N.B. arrows do not appear on the app but are shown here to display the direction of graphic movement.

The optimal SDB frequency is widely regarded in the extant literature to be ∼6 breaths min^−1^ (Cullins et al., 2013; Russo et al., 2017); accordingly, the third SDB condition was a fixed frequency of 6 breaths min^−1^ (6F*f*_r_). Both the dynamic algorithm and the fixed 6 breaths min^−1^ conditions were delivered by an app that provided visual feedback, via an iPad screen, which guided the user's breathing frequency. The user was instructed to inhale when the dome graphic rose and to exhale when the dome fell (Figure 1).

### 2.4 Data measurement and acquisition

Participants were seated in an upright position, at an approximate angle of 60° for the duration of the data collection. Respiratory airflow was monitored continuously throughout each breathing condition. Participants wore an oronasal mask that covered both mouth and nose (Oro Nasal 7450 V2 Mask, Hans Rudolph Inc., Shawnee, KS, USA) and respired flow rate was measured continuously using a heated pneumotachograph (Model 3700, Hans Rudolph) connected to a flow measurement system (RSS 100‐HR, Hans Rudolph).

Heart rate (*f*_c_) was monitored continuously using a three‐lead ECG and non‐invasive beat‐to‐beat arterial BP was estimated using a Finometer (Finapres NOVA, Finapres Medical Systems, Enschede, The Netherlands). The finger cuff‐derived BP was calibrated using an arm cuff prior to and halfway through data collection. Stroke volume (SV) was calculated by the Finometer using the Modelflow method (Wesseling et al., 1993). Total peripheral resistance (TPR) was calculated as mean arterial pressure divided by cardiac output ($\dot{Q}$). Peripheral pulse transit time (PTT) was calculated from the time delay between the peak of the R‐wave of the ECG and the peak of the pressure pulse recorded at the finger. End‐tidal CO_2_ was recorded at the end of each minute using an iWorx CO_2_/O_2_ Gas Analyser (GA‐200, iWorx Systems Inc., Dover, NH, USA).

Analog outputs from the Finapres NOVA (reconstructed brachial pressure waveform, ECG waveform, SV, SBP, DBP) and the respiratory flow meter were sampled continuously at 250 Hz via an analog to digital converter (NI USB‐6218 BNC, National Instruments Inc., Austin, TX, USA) and captured using acquisition and analysis software (LabView 2015, National Instruments, Inc.). The LabView software corrected for the 4 s delay between the Finapres NOVA output and the respiratory output. Data were recorded during the baseline period (5 min), and during each breathing condition (10 min; S*f*_r_, R*f*_r_, 6F*f*_r_, D*f*_r_).

Stretch stature was measured using a stadiometer (SECA 213, Seca, Hamburg, Germany) and body mass was recorded in minimal clothing using calibrated electronic scales (SECA 804, Germany).

### 2.5 Data analysis

Within the LabView software, cardiovascular and respiratory parameters were derived breath‐by‐breath, and minimum, maximum and mean values were calculated for every inspiration and expiration. Data were averaged in segments of 1 min, as well as mean values for the first 5 min, final 5 min, and the full 10 min for each condition. Data were compared for the three SDB conditions (R*f*_r_, 6F*f*_r_, D*f*_r_) and spontaneous breathing (S*f*_r_).

Respiratory sinus arrhythmia (RSA) was calculated using two methods across each breath phase: (1) the difference between the average heart rate (*f*_c_) during inspiration (*f*_c,i_) and expiration (*f*_c,e_) for every breath (*f*_c,Δ_); (2) the difference in maximum and minimum beat‐to‐beat intervals (RR) during inspiration and expiration, respectively, for every breath (RSA).

Because the kinetics of the haemodynamic perturbations created by breathing may lag the breathing phase in which they were generated, the ‘peak–valley’ method was used to analyse all cardiovascular variables; in other words, the difference between successive peaks (highest value) and valleys (lowest value) was calculated, independent of the breathing phase in which they occurred.

**FIGURE 2:**
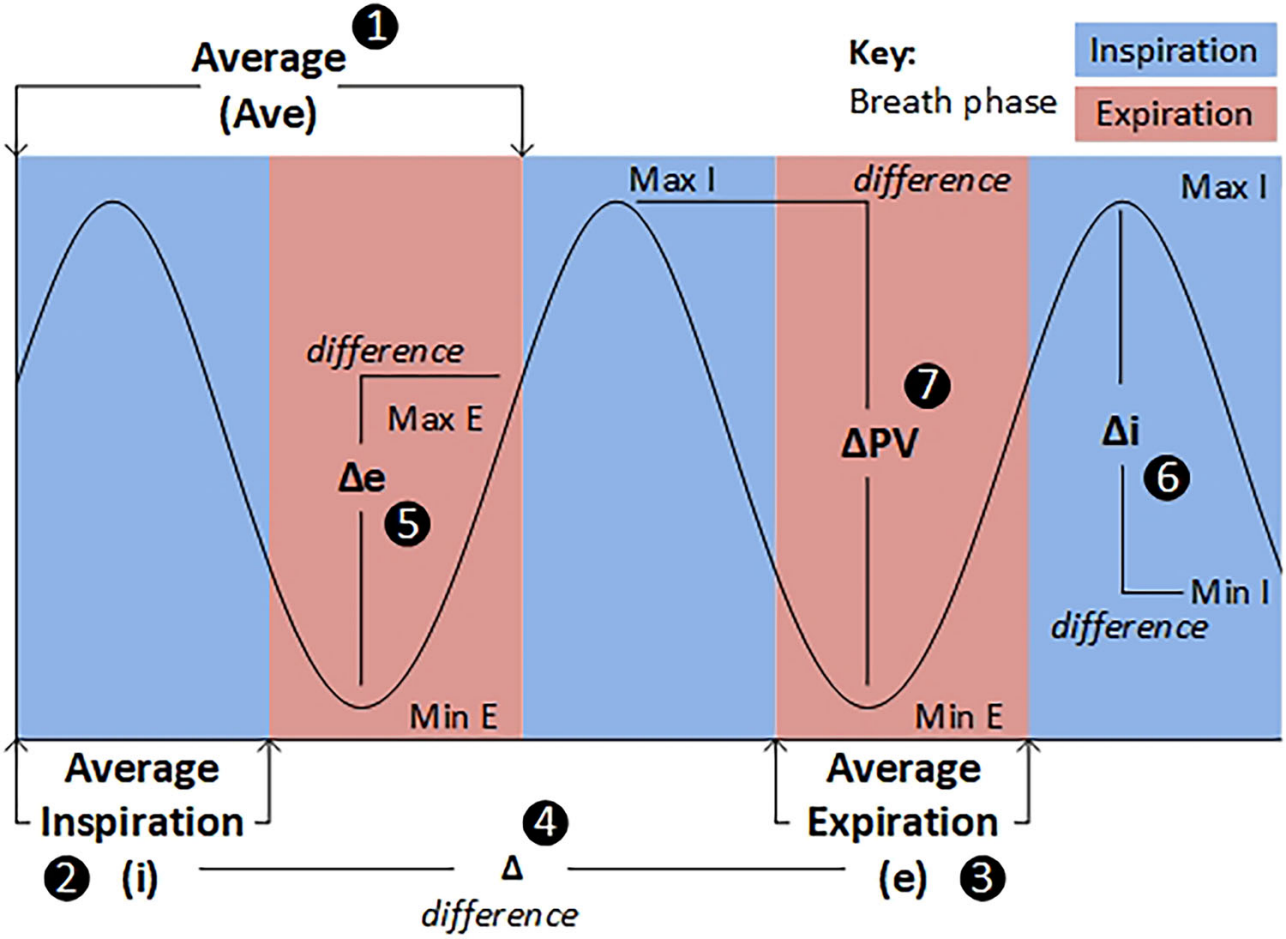
Calculations for example cardiovascular variable plot. E = Expiration breath phase; I = Inspiration breath phase. (1) Ave =average of whole breath; (2) i = average inspiration; (3) e =average expiration; (4) Δ = i minus e (average inspiration minus average expiration); (5) Δe = Max E minus Min E; (6) Δi = Max I minus Min I; (7) ΔPV = max I minus min E (note ΔPV calculation varies and can be Min I minus Max E depending on which calculation provides largest difference).

Calculated parameters and their derivations are displayed schematically using a sinewave in Figure 2 (with corresponding calculation numbers). Inter‐breath phase indices (Δ) were quantified as the difference between mean inspiration (i) and mean expiration (e) values for all variables (calculation 4). Peak–valley (PV) indices were calculated as maximum minus minimum values during inspiration (Δi: calculation 6) and during expiration (Δe: calculation 5). To represent the largest change in variables within breath phase, inter‐breath phase PV indices (ΔPV) were calculated using maximum inspiration minus minimum expiration, or minimum inspiration minus maximum expiration, dependent on which calculation gave the largest difference. Calculation 7 shows an example using the calculation maximum inspiration (peak) minus minimum expiration (valley). The PV indices were also calculated irrespective of the breath phase in which they occurred, and are referred to herein as peak–valley breath phase‐independent calculations (ΔPV_Ind; not shown in Figure 2).

Each condition was 10 min in duration, but the final 5 min segment of each SDB condition (R*f*_r_, 6F*f*_r_, D*f*_r_) were used for analysis to ensure relatively steady state values were analysed. For spontaneous breathing (S*f*_r_), the first 5 min segment was used, as participants were already in a steady state having rested prior to the condition for a minimum of 10 min while breathing normally/spontaneously. Dynamic breathing frequencies were also compared across the full 10 min condition and between the first and final 5 min.

Values are expressed as means ± SD unless stated otherwise. Statistical analysis was undertaken using SPSS Statistics 24 (IBM Corp., Armonk, NY, USA). After normality was confirmed for cardiovascular variables, repeated measures ANOVA with planned *post hoc* pairwise comparisons using Bonferroni corrections were used to compare between the different breathing frequencies. Reported *P*‐values are those following adjustment for repeated comparisons. For all analyses, *P* was set at 0.05. Correlation coefficients were calculated using Pearson's product moment.

## 3 RESULTS

Data were collected from 12 participants, but 1 participant was excluded due to failure to adhere to the prescribed breathing conditions (Table 1). Due to missing data from the S*f*_r_ condition for 2 participants, data from baseline spontaneous measurements were used in place of S*f*_r_ data for these 2 participants. Before doing so, data integrity checks were performed to ensure the substitution did not affect the study results. For all other participants (*n* = 9), it was confirmed that breathing frequency was not significantly different between baseline and the first 5 min S*f*_r_ condition. Additionally, there were no significant differences across variables between the baseline data and the first 5 min S*f*_r_ condition. There were no significant differences between males and females across all variables, so for clarity and to maintain a larger sample size, grouped data is presented. Sex disaggregated data can be found in Appendix A.

**TABLE 1:** Participant characteristics.

Characteristic	Value (*n* = 11)
Age (years)	41.3 ± 12.4
Stature (m)	1.7 ± 0.1
Mass (kg)	73.3 ± 9.9
BMI (kg/m^2^)	25.4 ± 4.2
Baseline SBP (mmHg)	118.2 ± 9.7
Baseline DBP (mmHg)	71.1 ± 9.3
Baseline *f*_r_ (breaths min^−1^)	12.2 ± 2.7
Baseline tidal volume (L)	0.6 ± 0.1

Data represent means ± SD (*n* = 11). Abbreviations: BMI, body mass index; DBP, diastolic blood pressure; *f*_r_, breathing frequency; SBP, systolic blood pressure.

### 3.1 Respiratory variables

**FIGURE 3:**
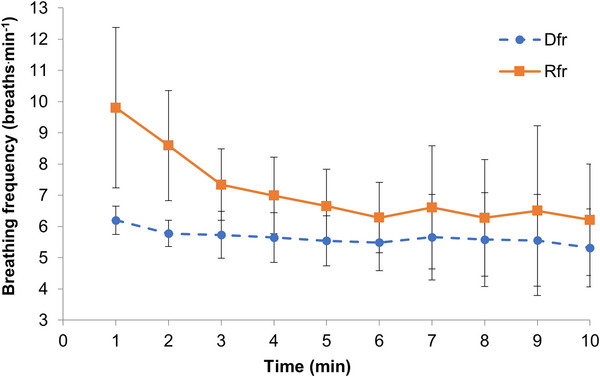
Breathing frequency during RESPeRATE (R*f*_r_) and dynamic breathing frequency (D*f*_r_) conditions. Data represent means ± SD (*n* = 11); solid line, RESPeRATE (R*f*_r_); dashed line, dynamic algorithm (D*f*_r_). Data points represent the average value for the preceding minute (1 min segment), i.e. data point at 5 min represents average breathing frequency between 4 and 5 min.

Table 2 provides an overview of the respiratory parameters for each condition. Breathing frequency during S*f*_r_ was significantly higher compared with all SDB conditions (R*f*_r_ *P* = 0.008; 6F*f*_r_ *P* = 0.003; D*f*_r_ *P* = 0.001), but was not significantly different between SDB conditions. The dynamic algorithm (D*f*_r_) computed the optimal breathing frequency (producing maximal RSA) to be 5.5 ± 1.3 breaths min^−1^ and the algorithm maintained a steady breathing frequency throughout the 10 min, with no difference in average breathing frequency between the first 5 min and final 5 min. The RESPeRATE (R*f*_r_) breathing frequency was 6.4 ± 1.9 breaths min^−1^ during the final 5 min, but produced a significantly higher breathing frequency during the first 5 min (Figure 3; 8.1 breaths min^−1^; *P* = 0.02). There was no significant difference in end‐tidal CO_2_ between any conditions (Table 2).

**TABLE 2:** Respiratory parameters.

	S*f*_r_	R*f*_r_	6F*f*_r_	D*f*_r_	Effect of condition *P*‐value
*f*_r_ (breaths min^−1^)	12.2 ± 3.7^bc^ *^d^*	6.4 ± 1.9^a^	6.0 ± 0.0^a^	5.5 ± 1.3^a^	<0.001
*V*_T_ (L)	0.6 ± 0.2^bc^ *^d^*	1.1 ± 0.4^a^	0.9 ± 0.3^a^	1.1 ± 0.4^a^	<0.001
*T*_I_/*T*_TOT_	0.4 ± 0.0	0.5 ± 0.1	0.6 ± 0.3	0.5 ± 0.0	0.129
End‐tidal CO_2_ (%)	5.0 ± 0.6	4.8 ± 0.7	5.0 ± 0.6	5.0 ± 0.7	0.535

Data represent means ± SD (*n* = 11). Significantly different from ^a^S*f*_r_. ^b^R*f*_r_. ^c^6*f*_r_. ^d^Df_r_.*P *< 0.05. Abbreviations: 6F*f*_r_, 6 breaths min^−^^1^; D*f*_r_, optimisation algorithm dynamic breathing frequency; *f*_r_, breathing frequency; R*f*_r_, RESPeRATE; S*f*_r_, spontaneous breathing; *T*_I_/*T*_TOT_, duty cycle; *V*_T_, tidal volume.

### 3.2 Peak–valley (ΔPV) and peak–valley breath phase‐independent (ΔPV_Ind)

**FIGURE 4:**
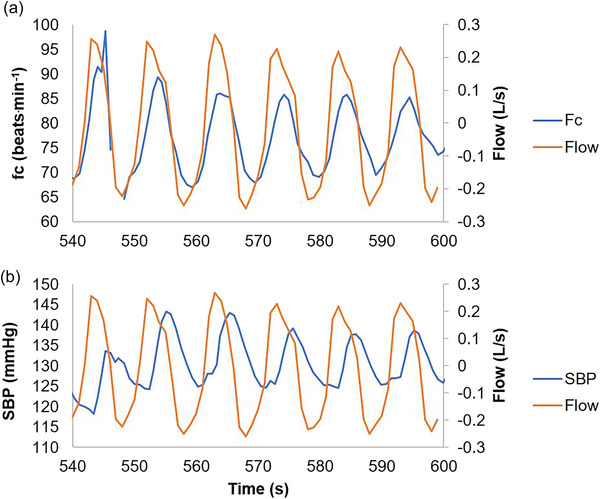
Respiratory synchronisation of heart rate (*f*_c_) (a) and systolic blood pressure (SBP) (b). Data for 1 participant during last minute of 6F*f*_r_ condition (6 breaths min^−1^). Respiratory flow (L/s: 1 s average). *f*_c_, heart rate (beats min^−1^); SBP, systolic blood pressure (mmHg).

Comparison of peak–valley values (ΔPV; highest difference between min/max inspiration and expiration; Calculation 7, Figure 2) and peak–valley breath phase‐independent values (ΔPV_Ind; highest difference across breath, irrespective of breath phase) reveals a clear difference in magnitude for some variables, such as SBP. As an example, Figure 4 shows the final minute of the 6F*f*_r_ condition for one participant; there was synchronisation between respiratory flow and heart rate in terms of both the variable and time/phase (Figure 4a), but asynchrony between respiratory flow and BP (Figure 4b) in terms of a constant time/phase lag for BP following behind respiratory flow. As such, when peak–valley calculations are analysed, larger amplitude differences are revealed when breath phase is disregarded. These are referred to henceforth as breath phase‐independent variables: ΔPV_Ind.

### 3.3 Arterial blood pressures

There were no significant differences for average SBP or DBP between breathing conditions (see Appendix B, Table B1), but the peak–valley amplitudes were significantly lower for S*f*_r_, compared with all SDB conditions (Table 3). All SDB conditions were significantly higher compared with S*f*_r_ for SBP_Δi_ (R*f*_r_ *P* = 0.003; 6F*f*_r_ *P* = 0.004; D*f*_r_ *P* = 0.001), and SBP_Δe_ (R*f*_r_ *P* = 0.031; 6F*f*_r_ *P *< 0.001; D*f*_r_ *P* = 0.007), and between S*f*_r_ and D*f*_r_ (*P* = 0.02) and 6F*f*_r_ (*P* = 0.011) for SBP_ΔPV_Ind_. These differences were replicated in the analogous DBP values. Peak–valley breath phase‐independent values (ΔPV_Ind) revealed larger SDB‐induced perturbations in SBP and DBP than peak–valley values (ΔPV).

**TABLE 3:** Peak–valley differences for blood pressure variables (mmHg).

	S*f*_r_	R*f*_r_	6F*f*_r_	D*f*_r_	Effect of condition *P*‐value
		6.4 ± 1.9			
SBP_Δi_ (mmHg)	3.4 ± 2.1^bcd^	10.9 ± 4.4^a^	10.0 ± 3.7^a^	11.4 ± 3.9^a^	<0.001
SBP %Δi	2.9%^bc^ *^d^*	9.5%^a^	8.8%^a^	10.2%^a^	<0.001
SBP_Δe_ (mmHg)	4.2 ± 2.4^bcd^	8.6 ± 4.5^a^	8.8 ± 3.3^a^	10.0 ± 4.8^a^	<0.001
SBP %Δe	3.4%^bcd^	7.2%^a^	7.3%^a^	8.4%^a^	<0.001
SBP_ΔPV_ (mmHg)	−8.1 ± 3.9	−10.2 ± 13.1	−10.3 ± 13.2	−14.4 ± 10.4	0.267
SBP_ΔPV_Ind_ (mmHg)	12.9 ± 3.3^cd^	16.0 ± 4.9	17.3 ± 4.3^a^	17.4 ± 6.5^a^	0.001
DBP_Δi_ (mmHg)	1.5 ± 0.9^bcd^	6.1 ± 2.9^a^	5.8 ± 2.5^a^	6.6 ± 2.5^a^	<0.001
DBP %Δi	2.1%^bcd^	9.0%^a^	8.2%^a^	9.6%^a^	<0.001
DBP_Δe_ (mmHg)	2.4 ± 1.1^cd^	5.1 ± 2.7	5.5 ± 2.9^a^	5.4 ± 2.3^a^	0.001
DBP %Δe	3.3%^bc^ *^d^*	7.2%^a^	7.7%^a^	7.6%^a^	0.002
DBP_ΔPV_ (mmHg)	−2.9 ± 2.3	−3.7 ± 8.4	−2.4 ± 8.5	−6.2 ± 6.4	0.292
DBP_ΔPV_Ind_ (mmHg)	7.0 ± 1.3^cd^	9.0 ± 2.7	9.3 ± 2.3^a^	9.3 ± 1.9^a^	0.007

Data represent means ± SD (*n* = 11).Significantly different from ^a^S*f*_r_. ^b^R*f*_r_. ^c^6*f*_r_. ^d^D*f*_r_. *P *< 0.05. Abbreviations: 6F*f*_r_, 6 breaths min^−^^1^; D*f*_r_, optimisation algorithm dynamic breathing frequency; DBP, diastolic blood pressure; R*f*_r_, RESPeRATE; SBP, systolic blood pressure; S*f*_r_, spontaneous breathing; Δe, within expiration difference; Δi, within inspiration difference; ΔPV, inter‐breath phase peak–valley difference; ΔPV_Ind, breath phase‐independent peak–valley difference; %Δe, percentage relative intra‐breath phase peak–valley differences during expiration; %Δi, percentage relative intra‐breath phase peak–valley differences during inspiration.

**FIGURE 5:**
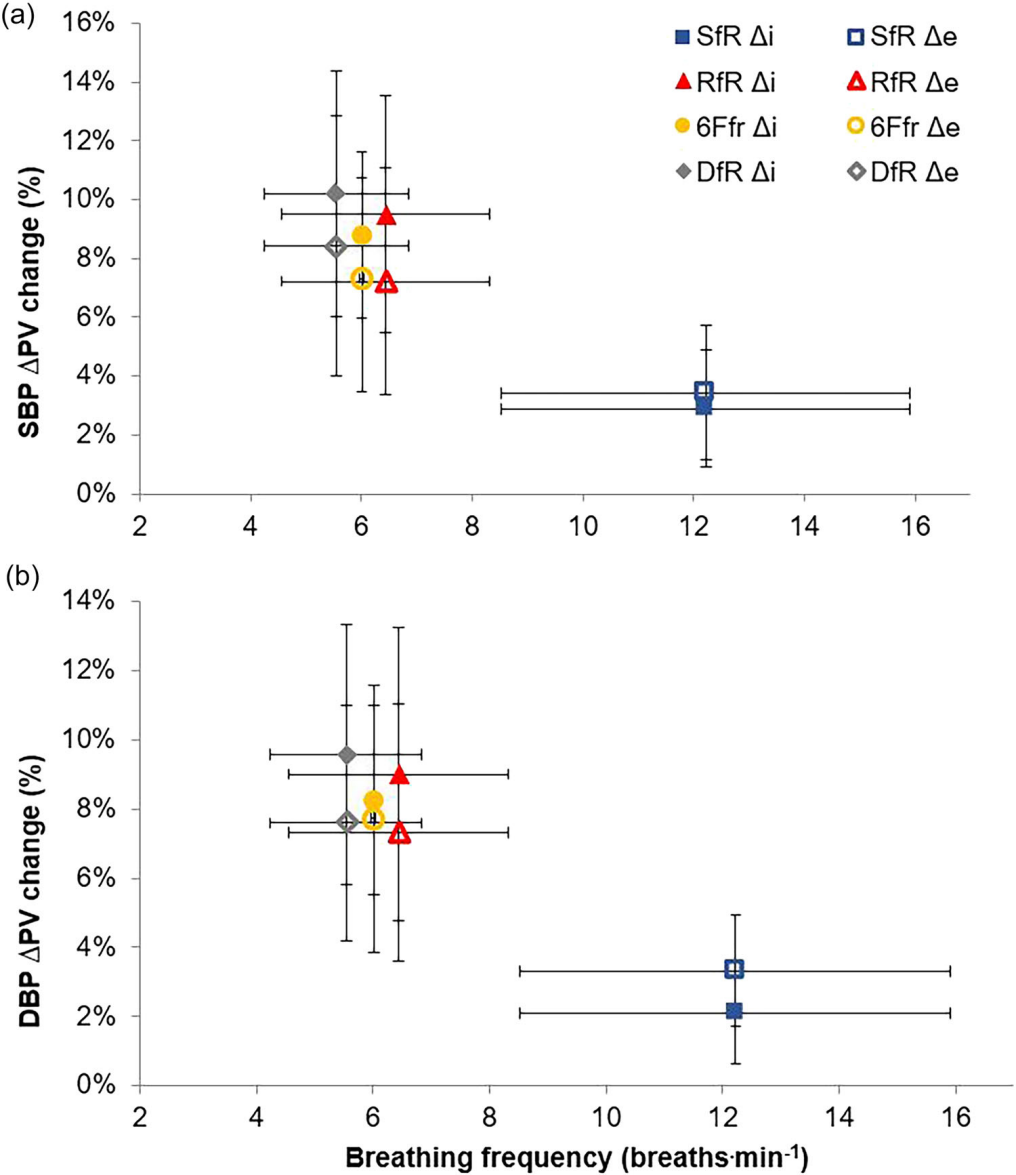
Blood pressure oscillations: %ΔI and %ΔE for systolic blood pressure (a) and diastolic blood pressure (b). Data represent means ± SD (*n* = 11). Variable calculated as SBP_∆i_ as a percentage of average SBP during inspiration, or equivalent during expiration and for DBP. DBP, diastolic blood pressure; SBP, systolic blood pressure; %∆i, within inspiration difference; %∆e, within expiration difference; S*f*_r_, spontaneous breathing; R*f*_r_, RESPeRATE, 6F*f*_r_, 6 breaths min^−1^; D*f*_r_, optimisation algorithm dynamic breathing frequency.

There were high correlations (>0.8) between SBP_Δi_ and SBP and between SBP_Δe_ and SBP and the DBP equivalents across all breathing conditions (see Appendix B, Table B2). Therefore, a percentage change in BP oscillations was calculated during inspiration and expiration, producing relative intra‐breath phase peak–valley differences (%Δi and %Δe; Table 3). The percentage BP oscillations were significantly larger for all SDB variables, compared with S*f*_r_ (Figure 5a, b), but produced no differences between SDB conditions. There were also significant differences for SBP %Δi (*P* = 0.026), SBP %Δe (*P* = 0.039) and DBP %Δi (*P* = 0.03) between the first 5 min and final 5 min for R*f*_r_, but only for SBP %Δi during the D*f*_r_ condition (*P* = 0.034), with a larger amplitude of fluctuations in the final 5 min for all variables.

### 3.4 Heart rate and respiratory sinus arrhythmia

**FIGURE 6:**
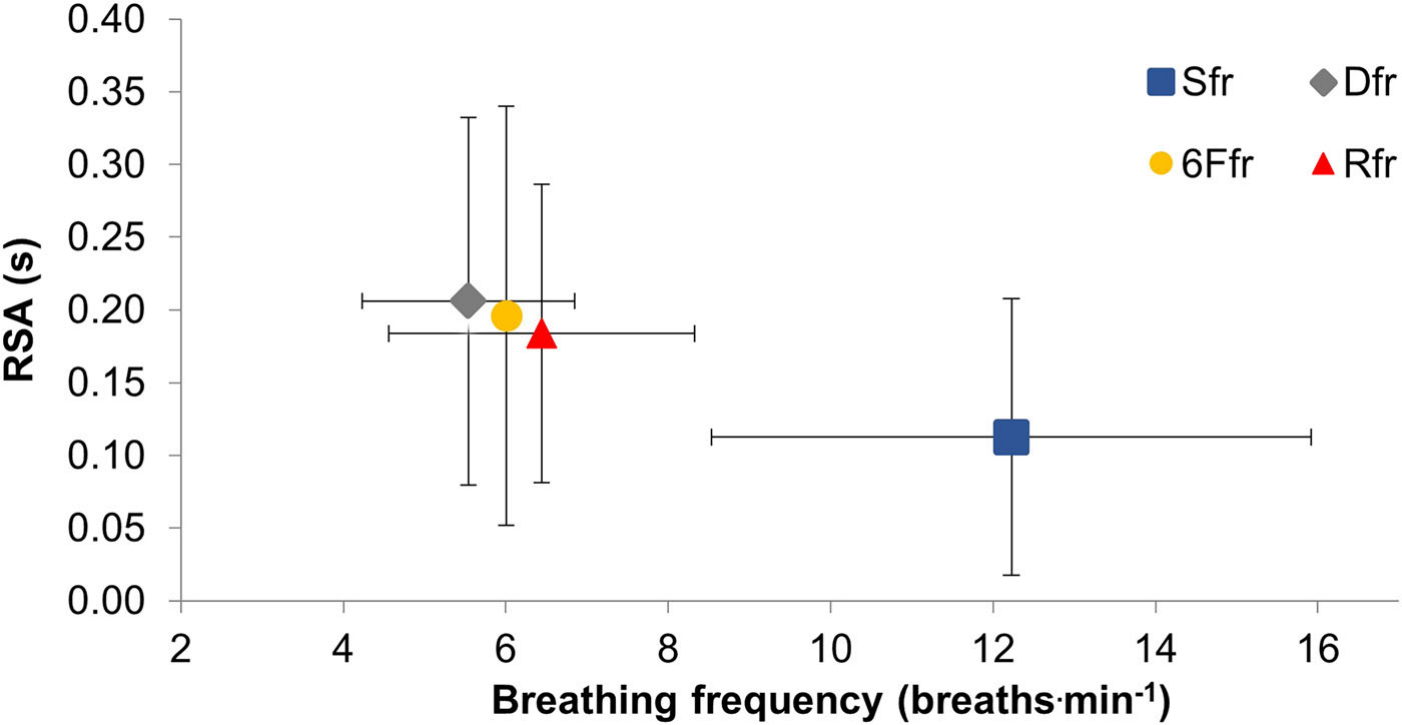
Respiratory sinus arrhythmia (RSA) response to slow and deep breathing. Data represent means ± SD (*n* = 11). S*f*_r_, spontaneous breathing; R*f*_r_, RESPeRATE, 6F*f*_r_, 6 breaths min^−1^; D*f*_r_, optimisation algorithm dynamic breathing frequency; RSA, respiratory sinus arrhythmia (s).

Average heart rate was significantly higher during 6F*f*_r_ (*P* = 0.003) and D*f*_r_ (*P* = 0.045), compared with S*f*_r_, but not during R*f*_r_ (*P* = 0.131; S*f*_r_ 58.6 ± 8.5; R*f*_r_ 60.6 ± 8.5; 6F*f*_r_ 62.4 ± 9.0; D*f*_r_ 62.3 ± 9.4 beats min^−1^; see Appendix B, Table B3 for full data set), whereas R*f*_r_ (*P* = 0.016) and 6F*f*_r_ (0 = 0.023) were significantly higher compared with S*f*_r_ for *f*_c,Δi_ (Table 4). Additionally, the amplitude of RSA was significantly lower during S*f*_r_ than during R*f*_r_ (*P *= 0.05) and D*f*_r_ (*P *= 0.018), but not for 6F*f*_r_ (*P *= 0.130; Figure 6); there were no differences between SDB conditions.

**TABLE 4:** Mean peak–valley differences for heart rate (*f*_c_) and respiratory sinus arrhythmia (RSA).

	S*f*_r_	R*f*_r_	6F*f*_r_	D*f*_r_	Effect of condition *P*‐value
		6.4 ± 1.9			
*f*_c,Δi_ (beats min^−^^1^)	3.6 ± 2.8^bc^	9.2 ± 5.1^a^	11.5 ± 6.8^a^	11.7 ± 8.3	0.004
*f*_c,Δe_ (beats min^−^^1^)	5.0 ± 3.5^c^	7.1 ± 3.7^c^	11.0 ± 5.6^ab^	10.0 ± 4.9	<0.001
*f*_c,ΔPV_ (beats min^−^^1^)	−1.6 ± 7.1	10.4 ± 8.9	9.3 ± 14.1	13.7 ± 10.7	0.021
RSA (s)	0.11 ± 0.10^b^ *^d^*	0.18 ± 0.10^a^	0.20 ± 0.14	0.21 ± 0.13^a^	0.001

Data represent means ± SD (*n* = 11). Significantly different from ^a^S*f*_r_. ^b^R*f*_r_. ^c^6*f*_r_. ^d^D*f*_r_. *P *< 0.05.

Abbreviations: 6F*f*_r_, 6 breaths min^−^^1^; D*f*_r_, optimisation algorithm dynamic breathing frequency; *f*_c_, heart rate; R*f*_r_, RESPeRATE; RSA, respiratory sinus arrhythmia; S*f*_r_, spontaneous breathing; Δe, within expiration difference; Δi, within inspiration difference; ΔPV, inter‐breath phase peak–valley difference.

### 3.5 Stroke volume and cardiac output

There was a significant effect of condition upon SV_∆i_ and SV_∆e_, but paired comparisons revealed no significant differences between breathing conditions (Table 5). Intra‐breath phase cardiac output ($\dot{Q}$) increased during SDB significantly and was significantly higher compared with S*f*_r_ for 6F*f*_r_ for ∆i (*P* = 0.023) and ∆e (*P* = 0.021), and for D*f*_r_ for ∆i (*P* = 0.038). Mean and breath phase mean data can be viewed in Appendix B, Table B4.

**TABLE 5:** Mean peak–valley differences for stroke volume (SV) and cardiac output ($\dot{Q}$).

	S*f*_r_	R*f*_r_	6F*f*_r_	D*f*_r_	Effect of condition *P*‐value
					
SV_Δi_ (mL)	5.3 ± 1.8	9.1 ± 6.1	10.3 ± 5.5	10.2 ± 5.6	0.006
SV_Δe_ (mL)	6.2 ± 2.4	8.9 ± 4.0	8.7 ± 2.8	9.8 ± 5.2	0.025
SV_ΔPV_ (mL)	−10.5 ± 3.4	−14.2 ± 7.1	−12.8 ± 11.9	−15.3 ± 6.9	0.384
SV_ΔPV_Ind_ (mL)	12.8 ± 3.4	12.6 ± 5.8	14.4 ± 7.1	13.6 ± 5.6	0.440
*Q̇*_Δi_ (mL min^−^^1^)	340.6 ± 234.2^c^ *^d^*	904.9 ± 583.0	1054.0 ± 602.1^a^	1077.4 ± 677.3^a^	<0.001
*Q̇*_Δe_ (mL min^−^^1^)	473.6 ± 333.8^c^	760.0 ± 391.2	933.1 ± 391.2^a^	854.9 ± 476.6	<0.001
*Q̇*_ΔPV_ (mL min^−^^1^)	−628.0 ± 571.1	363.8 ± 1147.4	105.7 ± 1392.5	277.8 ± 1299.4	0.083
*Q̇*_ΔPV_Ind_ (mL min^−^^1^)	918.1 ± 285.9	1070.2 ± 514.5	1267.7 ± 593.2	1135.7 ± 665.9	0.037

Data represent means ± SD (*n* = 11). Significantly different from ^a^S*f*_r_. ^b^R*f*_r_. ^c^6*f*_r_. ^d^D*f*_r_. *P *< 0.05. Abbreviations: 6F*f*_r_, 6 breaths min^−^^1^; D*f*_r_, optimisation algorithm dynamic breathing frequency; *Q̇*, cardiac output; R*f*_r_, RESPeRATE; S*f*_r_, spontaneous breathing; SV, stroke volume; Δe, within expiration difference; Δi, within inspiration difference; ΔPV, inter‐breath phase peak–valley difference; ΔPV_Ind, breath phase‐independent peak–valley difference.

### 3.6 Total peripheral resistance and pulse transit time

In keeping with the pattern of haemodynamic responses, intra‐breath phase total peripheral resistance (TPR) and peripheral transit time (PTT) increased during both phases of respiration (Table 6). Mean and breath phase mean data can be viewed in Appendix B, Table B5.

**TABLE 6:** Mean peak–valley differences for total peripheral resistance (TPR) and pulse transit time (PTT).

	S*f*_r_	R*f*_r_	6F*f*_r_	D*f*_r_	Effect of condition *P*‐value
					
TPR_Δi_ (mmHg min L^−^^1^)	1.5 ± 1.0^c^	2.7 ± 1.4	3.2 ± 1.9^a^	3.2 ± 1.8	0.001
TPR_Δe_ (mmHg min L^−^^1^)	2.0 ± 1.3	3.0 ± 1.6	3.5 ± 1.9	3.4 ± 2.4	0.004
TPR_ΔPV_ (mmHg min L^−^^1^)	1.0 ± 3.3*^d^*	−1.8 ± 3.9	−1.4 ± 4.7	−3.6 ± 3.7^a^	0.037
TPR_ΔPV_Ind_ (mmHg min L^−^^1^)	3.9 ± 1.6	3.9 ± 1.5	4.4 ± 2.1	4.2 ± 2.3	0.190
PTT_Δi_ (ms)	10 ± 6^c^	16 ± 7	19 ± 9^a^	21 ± 13	<0.001
PTT_Δe_ (ms)	12 ± 6^bc^	19 ± 10^a^	23 ± 12^a^	23 ± 17	0.001
PTT_ΔPV_ (ms)	16 ± 8	16 ± 17	21 ± 23	16 ± 29	0.750
PTT_ΔPV_Ind_ (ms)	20 ± 9	19 ± 8	26 ± 11	24 ± 14	0.076

Data represent means ± SD (*n* = 11). Significantly different from ^a^S*f*_r_. ^b^R*f*_r_. ^c^6*f*_r_. ^d^D*f*_r_. *P *< 0.05. Abbreviations: 6F*f*_r_, 6 breaths min^−^^1^; D*f*_r_, optimization algorithm dynamic breathing frequency; PTT, pulse transit time; R*f*_r_, RESPeRATE; S*f*_r_, spontaneous breathing; TPR, total peripheral resistance; Δe, within expiration difference; Δi, within inspiration difference; ΔPV, inter‐breath phase peak–valley difference; ΔPV_Ind_, breath phase‐independent peak–valley difference.

## 4 DISCUSSION

The main aim of the study was to characterise the multi‐parametric response to SDB using the AHA‐recommended RESPeRATE device and two other approaches to implementing SDB, including a comparison of responses between breathing conditions. This is the first study to provide a comprehensive characterisation of the acute cardiovascular responses to SDB, specifically analysing both inter‐ and intra‐breath perturbations created by breathing.

### 4.1 Effect of inter‐ and intra‐breath analysis

Our novel analysis highlights the importance of measuring and comparing more than simple average values, since only average heart rate showed a significant difference between spontaneous breathing and any methods of SDB (see Appendix B). Previous research has been limited and potentially misleading by its reliance upon average values, which as our data indicate, obscure the more complex cardiovascular oscillations created by SDB. However, more recent research supports this method, finding no differences in average heart rate values, and suggesting that RSA is the best method to measure parasympathetic changes caused by SDB (Ali et al., 2023). Our novel analysis provides evidence that differences between SDB and spontaneous breathing are only revealed by the peak–valley (Δi, Δe, ΔPV) and peak–valley breath phase‐independent (ΔPV_Ind) analyses. Therefore, analysis of inter‐ and intra‐breath oscillations is essential to reveal the full range of cardiovascular perturbations induced by SDB.

These perturbations are most prominently observed within the BP oscillations created by SDB, with all SDB conditions showing significant differences from spontaneous breathing in both inspiration and expiration and for SBP and DBP (Figure 5). The SBP oscillations within breath phases increased during SDB by up to 10.2% (11.4 mmHg) during inspiration (SBP_Δi_) and up to 8.4% (10 mmHg) during expiration (SBP_Δe_). In comparison, during spontaneous breathing (S*f*_r_) oscillations were less than half those induced by SDB, at just 2.9% (3.4 mmHg) and 3.4% (4.2 mmHg), respectively. For DBP, oscillations increased during SDB by up to 9.6% (6.6 mmHg) during inspiration and 7.7% (5.5 mmHg) during expiration, compared with fluctuations during S*f*_r_ of 2.1% (1.5 mmHg) and 3.3% (2.4 mmHg), respectively. Thus, SDB generates a profound and significant increase in the amplitude of BP oscillations, to which the baroreflex system will be subjected. Interestingly, the largest oscillations were found in the SDB condition with the lowest average breathing frequency (D*f*_r_). The amplitude of BP oscillations increased as breathing frequency was reduced and could perhaps be amplified further at breathing frequencies lower than those assessed in the present study. Extending breath phase duration allows more time for BP to fluctuate within‐breath, and provides a possible explanation for the largest fluctuations occurring during the slowest breathing frequency. Fluctuations in BP have been found previously and are potentially linked to cardiorespiratory coupling of respiration, BP and heart rate (Chang et al., 2013; Nuckowska et al., 2019; Russo et al., 2017). This is supported by the RSA data in the present study, which also increased as breathing frequency decreased, reaching a peak during D*f*_r_, the lowest breathing frequency. It may also be possible to further increase RSA, using frequencies lower than those used in the present study. However, since the dynamic algorithm was driven to maximise RSA, this seems less likely than for BP oscillations.

Additionally, during the SDB conditions the largest percentage within‐breath BP changes were observed during inspiration, but during spontaneous breathing the largest percentage change was during expiration. This reflects the known respiratory interactions where BP increases during inspiration when undertaking SDB, but decreases during inspiration during spontaneous breathing, so‐called pulsus paradoxus (Parati et al., 2008). The largest perturbations therefore occur in the breath phase in which BP is rising. During inspiration, negative intrathoracic pressure enhances venous return, which may be amplified by SDB due to larger amplitude changes of intra‐thoracic pressure (Russo et al., 2017), resulting from an increase in tidal volume when breathing frequency is lowered. The increased BP oscillations during inspiration may therefore be a reflection of the cardiovascular responses to the change in intra‐thoracic pressure and subsequent increased venous return during SDB.

### 4.2 Effect of response kinetics

A key finding from this study is the higher amplitude of ‘breath phase‐independent’ cardiovascular oscillations, as well as those of the peak–valley intra‐breath phase fluctuations. Figure 4 shows the differing synchronisation between respiratory flow and heart rate, compared with respiratory flow and SBP as an example using an individual participant. For heart rate across all participants, the peak–valley value (RSA) matches closely the peak–valley breath phase‐independent values. This occurs because of the faster kinetics of the heart rate response, leading to synchronisation of heart rate and breathing phase. In contrast, the oscillations of other variables, such as SBP, are misrepresented by inter‐breath phase peak–valley values; in Figure 4b the minimum and maximum SBP values occur during the same breath phase, which reflects the influence of differing kinetics of the effect of breathing upon heart rate and haemodynamics. If one only considers the instantaneous haemodynamic responses during a given breath phase, then the true amplitude of the perturbations created by SDB is obscured. This is reflected in our statistical analyses, as only ΔPV_Ind values, and not ΔPV, were significantly different between conditions for $\dot{Q}$, SBP and DBP. Therefore, to reveal the true cardiovascular perturbations, it is important to evaluate breath phase‐independent values of cardiovascular oscillations. Coherence analysis could further the understanding of this phenomenon, but was beyond the scope of this study. For a full review of coherence and slow and deep breathing see Sevoz‐Couche & Laborde (2022).

### 4.3 Comparison of SDB conditions

There were no significant differences between the responses induced by the three SDB conditions during the final 5 min. This suggests that the 6F*f*_r_ and D*f*_r_ conditions induced similar amplitudes of cardiovascular perturbation as the AHA‐recommended RESPeRATE, a device already shown to reduce BP when practised daily (Chaddha et al., 2019). From the perspective of the haemodynamic perturbations induced, it seems that the most important feature of SDB is that breathing frequency is ∼6 breaths min^−1^, rather than how this frequency is achieved. The physiological response to SDB seems not to be influenced by delivery method, including whether SDB is delivered with or without biofeedback (Laborde, Allen, Borges, Iskra et al., 2022). However, when considering the ΔPV_Ind values, only 6F*f*_r_ and D*f*_r_ were significantly different from S*f*_r_ for SBP_ΔPV_Ind_ and DBP_ΔPV_Ind_, suggesting they may generate slightly superior cardiovascular perturbations to RESPeRATE.

Since 6F*f*_r_ and D*f*_r_ produce the same cardiovascular response as RESPeRATE, it is reasonable to suggest they may produce the same anti‐hypertensive effect. Our data indicate that, at the very least, 6F*f*_r_ and D*f*_r_ provide alternative methods to implement SDB as an intervention to reduce BP. Indeed, 6F*f*_r_ and D*f*_r_ may prove superior to RESPeRATE, since the reduced breathing frequency (∼6 breaths min^−1^) is experienced for a longer duration (Figure 3), as the conditions either reduce breathing frequency faster (dynamic algorithm) or maintain the same reduced frequency throughout (6 breaths min^−1^); RESPeRATE produced an average frequency of 8.1 breaths min^−1^ during the first 5 min compared with 6.4 breaths min^−1^ in the last 5 min, whilst the dynamic algorithm produced a frequency of 5.8 breaths min^−1^ (first 5 min) and 5.5 breaths min^−1^ (last 5 min), respectively. Additionally, there were significantly higher BP oscillations during the final 5 min of RESPeRATE than the first 5 min, showing the potential for different acute cardiovascular responses at higher SDB frequencies. Further research is required to determine whether the haemodynamic responses at ∼8 breaths min^−1^ and ∼6 breaths min^−1^ differ, and whether any acute differences reflect superiority/inferiority in the anti‐hypertensive effect of SDB.

A final practical consideration is whether the increased ‘exposure time’ to the SDB frequencies delivered by the 6F*f*_r_ and D*f*_r_ conditions could shorten the length of the daily SDB intervention, compared with the recommended duration of RESPeRATE use. It is reasonable to suggest that if the stimulus (SDB at ∼6 breaths min^−1^) is attained more quickly than is the case when using the RESPeRATE device, then the overall duration of the SDB session could be reduced (e.g. reducing or 10 min to 5 min). Further research examining the long‐term benefits to BP of these alternative approaches to delivering SDB is needed to test this theory.

### 4.4 Potential restoration of autonomic imbalance by slow and deep breathing

When considering the mechanisms by which SDB may reduce chronic blood pressure, comparison with baroreflex activation therapy is pertinent. During SDB, baroreflex sensitivity has been observed to increase acutely (Lewis et al., 2018). It has been suggested that SDB stimulates the vagus nerve via its action on the baroreflex, which may result in chronic functional changes leading to a reduction of blood pressure (Laborde, Allen, Borges, Dosseville et al., 2022).

The present study has revealed an increase in the amplitude of fluctuations in BP during SDB, which may provide a similar mechanistic pathway to reduce BP as baroreflex activation therapy, by increasing activation of the baroreceptors due to the increased number and amplitude of BP changes. However, as baroreflex sensitivity was not measured in this study we cannot categorically conclude that the BP oscillations do produce an increase in baroreflex sensitivity.

### 4.5 Limitations

The equipment utilised to monitor haemodynamic changes was non‐invasive; consequently, all cardiovascular variables are estimates of the true responses. However, the Modelflow method used by the Finapres to estimate SV produces measurements that show excellent agreement with SV measured by Doppler ultrasound (Van Lieshout et al., 2003) and when blood is withdrawn by phlebotomy (Leonetti et al., 2004). Additionally, BP measures from the Finapres correlate highly with auscultatory BP measurements in normotensive participants (Carlson et al., 2019). Nonetheless, an acknowledgement of the use of indirect measurement is required, especially in the context of the accuracy of measurements during SDB.

### 4.6 Conclusion

In conclusion, all three SDB conditions elicited similar cardiovascular responses to each other, when compared with spontaneous breathing. Thus, it is reasonable to suggest that both an RSA‐driven dynamic algorithm (D*f*_r_) and a fixed frequency of 6 breaths min^−1^ (6F*f*_r_) may have similar anti‐hypertensive effects to the RESPeRATE device. Future research addressing the acute effects of SDB should explore a range of breathing frequencies to examine if BP oscillations can be maximised at breathing frequencies <6 breaths min^−1^ and whether SDB at higher frequencies of 8 breaths min^−1^ (replicating the first 5 min of RESPeRATE) produces the same acute cardiovascular responses as found in the present study. All future studies should note the importance of looking beyond average responses to examine inter‐ and intra‐breath phase cardiovascular oscillations, especially for BP, to reveal the full extent of the cardiovascular responses to SDB. In this respect, analysis of breath phase‐independent peak–valley fluctuations of cardiovascular variables seems most appropriate and pragmatic.

## AUTHOR CONTRIBUTIONS

The experiments were performed at Bournemouth University and author contributions are as follows. Conception or design of the work (Malika Felton); acquisition, analysis or interpretation of data for the work (Malika Felton, Vikram Mohan, Vanora A. Hundley); drafting the work or revising it critically for important intellectual content (Malika Felton, Vikram Mohan, Vanora A. Hundley). All authors have read and approved the final version of this manuscript and agree to be accountable for all aspects of the work in ensuring that questions related to the accuracy or integrity of any part of the work are appropriately investigated and resolved. All persons designated as authors qualify for authorship, and all those who qualify for authorship are listed.

## CONFLICT OF INTEREST

The authors have declared that no conflict of interest exist.

## Data Availability

The data that support the findings of this study are openly available in BORDaR at https://doi.org/10.18746/bmth.data.00000513.

## APPENDIX A

### A.1

The following results (Tables A1, A2, A3, A4, A5, A6, A7, A8, A9, A10) present the sex‐disaggregated data for mean values and peak–valley differences for all variables.

**TABLE A1:** Participant characteristics.

	Female	Male	*P*‐value
	(*n* = 6)	(*n *= 5)	
Age (years)	42.0 ± 10.1	40.4 ± 15.9	0.844
Stature (m)	1.66 ± 0.06	1.76 ± 0.04	0.013^*^
Mass (kg)	71.5 ± 10.9	75.4 ± 9.3	0.546
BMI (kg/m^2^)	26.2 ± 5.5	24.4 ± 2.3	0.500
Baseline SBP (mmHg)	118.3 ± 11.4	118.0 ± 8.6	0.958
Baseline DBP (mmHg)	72.2 ± 11.4	69.8 ± 7.0	0.696
Baseline *f*_r_ (breaths min^−1^)	12.5 ± 2.8	12.0 ± 2.8	0.750
Baseline tidal volume (L)	0.5 ± 0.2	0.6 ± 0.1	0.472

Data represent means ± SD (*n* = 11; female = 6, male = 5). ^*^Significant difference between groups. Abbreviations: BMI, body mass index; DBP, diastolic blood pressure; *f*_r_, breathing frequency; SBP, systolic blood pressure.

**TABLE A2:** Respiratory parameters.

		S*f*_r_	R*f*_r_	6F*f*_r_	D*f*_r_	Effect of condition *P*‐value	Sex × Condition *P*‐value
*f*_r_ (breaths min^−^^1^)	F	12.2 ± 4.7	6.0 ± 1.2	6.0 ± 0.1	5.8 ± 1.7	<0.001	0.735
	M	12.3 ± 2.5	7.0 ± 2.5	6.0 ± 0.0	5.2 ± 0.4		
	All	12.2 ± 3.7^bcd^	6.4 ± 1.9^a^	6.0 ± 0.0^a^	5.5 ± 1.3^a^		
*V*_T_ (L)	F	0.6 ± 0.2	1.2 ± 0.4	0.9 ± 0.5	1.1 ± 0.5	<0.001	0.621
	M	0.6 ± 0.1	1.0 ± 0.4	0.9 ± 0.2	1.1 ± 0.4		
		0.6 ± 0.2^bcd^	1.1 ± 0.4^a^	0.9 ± 0.3^a^	1.1 ± 0.4^a^		
*T*_I_/*T*_TOT_	F	0.4 ± 0.0	0.5 ± 0.1	0.5 ± 0.1	0.5 ± 0.1	0.129	0.569
	M	0.4 ± 0.0	0.5 ± 0.1	0.6 ± 0.4	0.5 ± 0.0		
	All	0.4 ± 0.0	0.5 ± 0.1	0.6 ± 0.3	0.5 ± 0.0		
End‐tidal CO_2_ (%)	F	4.7 ± 0.6	4.4 ± 0.7	4.7 ± 0.7	4.7 ± 0.8	0.535	0.167
	M	5.3 ± 0.5	5.1 ± 0.5	5.2 ± 0.5	5.3 ± 0.6		
	All	5.0 ± 0.6	4.8 ± 0.7	5.0 ± 0.6	5.0 ± 0.7		

Data represent means ± SD (All *n* = 11, female *n* = 6, male *n* = 5). Significantly different from ^a^S*f*_r_. ^b^R*f*_r_. ^c^6*f*_r_. ^d^D*f*_r_. *P *< 0.05. Abbreviations: 6F*f*_r_, 6 breaths min^−^^1^; D*f*_r_, optimization algorithm dynamic breathing frequency; F, female; *f*_r_, breathing frequency; M, male; R*f*_r_, RESPeRATE; S*f*_r_, spontaneous breathing; *T*_I_/*T*_TOT_, duty cycle; *V*_T_, tidal volume.

**TABLE A3:** Mean values for blood pressure variables.

		S*f*_r_	R*f*_r_	6F*f*_r_	D*f*_r_	Effect of condition *P*‐value	Sex × Condition *P*‐value
SBP (mmHg)	F	117.5 ± 16.8	115.9 ± 14.4	116.0 ± 15.5	115.0 ± 10.5	0.358	0.857
	M	123.0 ± 5.8	118.7 ± 7.3	118.3 ± 7.9	116.1 ± 10.4		
	All	120.0 ± 12.8	117.2 ± 11.2	117.1 ± 12.1	115.5 ± 9.9		
SBP_i_ (mmHg)	F	114.8 ± 16.9	114.2 ± 14.7	114.3 ± 15.4	112.6 ± 11.5	0.157	0.445
	M	121.3 ± 5.0	115.3 ± 8.2	113.7 ± 8.2	111.3 ± 11.6		
	All	117.7 ± 12.8	114.7 ± 11.6	114.0 ± 12.1	112.0 ± 11.0		
SBP_e_ (mmHg)	F	120.3 ± 16.8	117.6 ± 14.7	117.8 ± 16.2	117.5 ± 9.7	0.659	0.992
	M	124.8 ± 6.7	122.1 ± 7.0	122.9 ± 8.2	121.0 ± 9.7		
	All	122.3 ± 12.8	119.7 ± 11.5	120.1 ± 12.8	119.1 ± 9.4		
SBP_Δ_ (mmHg)	F	−5.5 ± 2.3	−3.4 ± 6.3	−3.5 ± 6.2	−4.9 ± 3.4	0.158	0.026
	M	−3.5 ± 2.1	−6.9 ± 4.5	−9.2 ± 4.6	−9.6 ± 5.1		
	All	−4.6 ± 2.3	−5.0 ± 5.6	−6.1 ± 6.0	−7.0 ± 4.7		
DBP (mmHg)	F	70.5 ± 10.3	70.0 ± 7.4	70.8 ± 10.5	70.4 ± 9.4	0.412	0.592
	M	72.6 ± 7.7	67.6 ± 8.8	70.7 ± 8.9	69.3 ± 9.8		
	All	71.4 ± 8.8	68.9 ± 7.7	70.7 ± 9.3	69.9 ± 9.1		
DBP_i_ (mmHg)	F	69.5 ± 10.2	70.0 ± 8.8	70.5 ± 11.6	69.8 ± 10.0	0.326	0.232
	M	72.4 ± 7.6	66.3 ± 8.8	68.9 ± 8.5	67.2 ± 9.8		
	All	70.8 ± 8.8	68.3 ± 8.6	69.8 ± 9.9	68.6 ± 9.5		
DBP_e_ (mmHg)	F	71.5 ± 10.5	70.0 ± 6.3	71.0 ± 9.6	71.0 ± 9.0	0.482	0.886
	M	72.8 ± 7.7	68.9 ± 8.9	72.4 ± 9.4	71.3 ± 9.8		
	All	72.1 ± 8.9	69.5 ± 7.2	71.7 ± 9.0	71.2 ± 8.9		
DBP_Δ_ (mmHg)	F	−1.9 ± 1.1	−0.1 ± 4.1	−0.5 ± 3.2	−1.2 ± 2.0	0.288	0.031
	M	−0.4 ± 0.6	−2.6 ± 2.0	−3.5 ± 1.8	−4.1 ± 2.4		
	All	−1.2 ± 1.2	−1.2 ± 3.4	−1.9 ± 3.0	−2.5 ± 2.5		

Data represent means ± SD (All *n* = 11, female *n* = 6, male *n* = 5). Abbreviations: 6F*f*_r_, 6 breaths min^−^^1^; DBP, diastolic blood pressure; D*f*_r_, optimization algorithm dynamic breathing frequency; e, mean expiration; F, female; i, mean inspiration; M, male; R*f*_r_, RESPeRATE; SBP, systolic blood pressure; S*f*_r_, spontaneous breathing; Δ, inter‐breath phase difference (i minus e).

**TABLE A4:** Peak–valley differences for blood pressure variables.

		S*f*_r_	R*f*_r_	6F*f*_r_	D*f*_r_	Effect of condition *P*‐value	Sex × Condition *P*‐value
SBP_Δi_ (mmHg)	F	3.6 ± 2.6	11.7 ± 3.5	10.5 ± 2.5	11.7 ± 4.5	<0.001	0.979
	M	3.0 ± 1.6	10.0 ± 5.6	9.5 ± 5.0	11.1 ± 3.6		
	All	3.4 ± 2.1^bc^ *^d^*	10.9 ± 4.4^a^	10.0 ± 3.7^a^	11.4 ± 3.9^a^		
SBP_Δe_ (mmHg)	F	4.7 ± 2.6	10.0 ± 2.4	10.6 ± 2.1	10.9 ± 4.3	<0.001	0.611
	M	3.5 ± 2.3	7.0 ± 6.1	6.6 ± 3.2	9.0 ± 5.7		
	All	4.2 ± 2.4^bcd^	8.6 ± 4.5^a^	8.8 ± 3.3^a^	10.0 ± 4.8^a^		
SBP_ΔPV_ (mmHg)	F	−9.2 ± 4.1	−6.8 ± 16.0	−5.4 ± 15.8	−11.5 ± 11.8	0.267	0.251
	M	−6.6 ± 3.5	−14.3 ± 8.6	−16.1 ± 6.7	17.9 ± 8.3		
	All	−8.1 ± 3.9	−10.2 ± 13.1	−10.3 ± 13.2	−14.4 ± 10.4		
SBP_ΔPV_Ind_ (mmHg)	F	13.4 ± 3.3	15.9 ± 3.3	17.2 ± 3.9	15.7 ± 5.2	0.001	0.150
	M	12.4 ± 3.6	16.2 ± 6.9	17.3 ± 5.1	19.5 ± 7.8		
	All	12.9 ± 3.3^cd^	16.0 ± 4.9	17.3 ± 4.3^a^	17.4 ± 6.5^a^		
DBP_Δi_ (mmHg)	F	1.9 ± 1.0	7.1 ± 2.9	7.2 ± 2.4	7.6 ± 2.8	<0.001	0.635
	M	1.0 ± 0.4	5.0 ± 2.8	4.1 ± 1.3	5.3 ± 1.5		
	All	1.5 ± 0.9^bcd^	6.1 ± 2.9^a^	5.8 ± 2.5^a^	6.6 ± 2.5^a^		
DBP_Δe_ (mmHg)	F	2.9 ± 1.1	6.1 ± 2.8	7.2 ± 2.7	6.4 ± 2.1	0.001	0.463
	M	1.7 ± 0.8	3.8 ± 2.1	3.5 ± 1.8	4.3 ± 2.3		
	All	2.4 ± 1.1^cd^	5.1 ± 2.7	5.5 ± 2.9^a^	5.4 ± 2.3^a^		
DBP_ΔPV_ (mmHg)	F	−4.2 ± 1.7	−1.4 ± 10.8	1.2 ± 10.3	−4.8 ± 8.3	0.292	0.096
	M	−1.2 ± 1.8	−6.5 ± 3.6	−6.7 ± 2.5	−8.0 ± 3.3		
	All	−2.9 ± 2.3	−3.7 ± 8.4	−2.4 ± 8.5	−6.2 ± 6.4		
DBP_ΔPV_Ind_ (mmHg)	F	7.7 ± 1.4	9.9 ± 3.1	10.6 ± 1.9	9.4 ± 1.3	0.007	0.288
	M	6.2 ± 0.2	7.8 ± 2.0	7.7 ± 1.6	9.2 ± 2.6		
	All	7.0 ± 1.3^cd^	9.0 ± 2.7	9.3 ± 2.3^a^	9.3 ± 1.9^a^		

Data represent means ± SD (All *n* = 11, female *n* = 6, male *n* = 5). Significantly different from ^a^S*f*_r_. ^b^R*f*_r_. ^c^6*f*_r_. ^d^D*f*_r_.  *P *< 0.05. Abbreviations: 6F*f*_r_, 6 breaths min^−^^1^; DBP, diastolic blood pressure; D*f*_r_, optimization algorithm dynamic breathing frequency; F, female; M, male; R*f*_r_, RESPeRATE; SBP, systolic blood pressure; S*f*_r_, spontaneous breathing; Δe, within‐expiration difference; Δi, within‐inspiration difference; ΔPV, inter‐breath phase peak–valley difference; ΔPV_Ind, breath phase‐independent peak–valley difference.

**TABLE A5:** Mean values for heart rate (*f*_c_).

		S*f*_r_	R*f*_r_	6F*f*_r_	D*f*_r_	Effect of condition *P*‐value	Sex × Condition *P*‐value
*f*_c_ (beats min^−^^1^)	F	63.6 ± 7.6	65.7 ± 8.0	67.6 ± 7.9	67.3 ± 8.8	<0.001	0.999
	M	52.5 ± 4.8	54.5 ± 3.8	56.3 ± 6.1	56.2 ± 6.2		
	All	58.6 ± 8.5^cd^	60.0 ± 8.5	62.4 ± 9.0^a^	62.3 ± 9.4^a^		
*f*_c,i_ (beats min^−^^1^)	F	63.6 ± 6.9	68.0 ± 8.7	69.3 ± 9.2	69.4 ± 9.0	<0.001	0.960
	M	52.0 ± 4.1	56.8 ± 3.4	58.5 ± 5.7	59.0 ± 6.2		
	All	58.4 ± 8.2^bcd^	62.9 ± 8.8^a^	64.4 ± 9.3^a^	64.7 ± 9.2^a^		
*f*_c,e_ (beats min^−^^1^)	F	63.8 ± 8.5	63.4 ± 7.6	66.0 ± 7.4	65.2 ± 8.7	0.125	0.921
	M	52.9 ± 5.7	52.2 ± 4.9	54.0 ± 7.6	53.3 ± 6.9		
	All	58.8 ± 9.0	58.3 ± 8.5	60.5 ± 9.5	59.8 ± 9.8		
*f*_c,Δ_ (beats min^−^^1^)	F	−0.1 ± 2.3	4.6 ± 3.1	3.3 ± 5.2	4.1 ± 2.4	0.005	0.865
	M	−0.9 ± 3.1	4.5 ± 3.6	4.5 ± 5.7	5.7 ± 4.2		
	All	−0.5 ± 2.6^bd^	4.6 ± 3.2^a^	3.8 ± 5.2	4.9 ± 3.3^a^		

Data represent means ± SD (All *n* = 11, female *n* = 6, male *n* = 5). Significantly different from ^a^S*f*_r_. ^b^R*f*_r_. ^c^6*f*_r_. ^d^D*f*_r_. *P *< 0.05. Abbreviations: 6F*f*_r_, 6 breaths min^−^^1^; D*f*_r_, optimization algorithm dynamic breathing frequency; e, mean expiration; F, female; *f*_c_, heart rate; i, mean inspiration; M, male; R*f*_r_, RESPeRATE; S*f*_r_, spontaneous breathing; Δ, inter‐breath phase difference (i minus e).

**TABLE A6:** Peak–valley differences for heart rate (*f*_c_) and respiratory sinus arrhythmia (RSA).

		S*f*_r_	R*f*_r_	6F*f*_r_	D*f*_r_	Effect of condition *P*‐value	Sex × Condition *P*‐value
*f*_c,Δi_ (beats min^−^^1^)	F	4.3 ± 2.5	10.9 ± 5.0	13.4 ± 7.5	13.6 ± 10.3	0.004	0.741
	M	2.6 ± 2.9	7.2 ± 4.8	9.3 ± 5.9	9.4 ± 5.3		
	All	3.6 ± 2.8^bc^	9.2 ± 5.1^a^	11.5 ± 6.8^a^	11.7 ± 8.3		
*f*_c,Δe_ (beats min^−^^1^)	F	6.5 ± 3.9	7.6 ± 4.3	11.6 ± 5.5	9.6 ± 3.7	<0.001	0.477
	M	3.2 ± 2.2	6.4 ± 3.2	10.3 ± 6.3	10.4 ± 6.5		
	All	5.0 ± 3.5^c^	7.1 ± 3.7^c^	11.0 ± 5.6*^a^* ^b^	10.0 ± 4.9		
*f*_c,ΔPV_ (beats min^−^^1^)	F	−2.1 ± 7.7	11.5 ± 10.6	8.2 ± 17.1	14.2 ± 13.3	0.021	0.963
	M	−1.1 ± 6.8	9.2 ± 7.4	10.6 ± 11.3	13.2 ± 8.1		
	All	−1.6 ± 7.1	10.4 ± 8.9	9.3 ± 14.1	13.7 ± 10.7		
RSA (s)	F	0.09 ± 0.04	0.16 ± 0.05	0.14 ± 0.08	0.15 ± 0.04	0.001	0.284
	M	0.13 ± 0.13	0.22 ± 0.15	0.26 ± 0.19	0.27 ± 0.17		
	All	0.11 ± 0.10^bd^	0.18 ± 0.10^a^	0.20 ± 0.14	0.21 ± 0.13^a^		

Data represent means ± SD (All *n* = 11, female *n* = 6, male *n* = 5). Significantly different from ^a^S*f*_r_. ^b^R*f*_r_. ^c^6*f*_r_. ^d^D*f*_r_. *P *< 0.05. Abbreviations: 6F*f*_r_, 6 breaths min^−^^1^; Δe, within‐expiration difference; Δi, within‐inspiration difference; ΔPV, inter‐breath phase peak–valley difference; D*f*_r_, optimization algorithm dynamic breathing frequency; F, female; *f*_c_, heart rate; M, male; R*f*_r_, RESPeRATE; RSA, respiratory sinus arrhythmia; S*f*_r_, spontaneous breathing.

**TABLE A7:** Mean values for stroke volume (SV) and cardiac output ($\dot{Q}$).

		S*f*_r_	R*f*_r_	6F*f*_r_	D*f*_r_	Effect of condition *P*‐value	Sex × Condition *P*‐value
SV (mL)	F	77.5 ± 14.7	74.8 ± 15.5	74.0 ± 13.8	75.1 ± 13.3	0.027	0.205
	M	82.0 ± 13.8	79.1 ± 15.4	79.6 ± 13.6	74.5 ± 14.0		
	All	79.6 ± 13.8	76.7 ± 14.8	76.6 ± 13.3	74.8 ± 12.9		
SV_i_ (mL)	F	75.3 ± 14.9	72.8 ± 15.4	72.4 ± 12.7	72.8 ± 13.0	0.013	0.232
	M	79.4 ± 14.3	76.4 ± 14.5	75.6 ± 13.3	71.4 ± 13.9		
	All	77.1 ± 14.0	74.4 ± 14.4	73.9 ± 12.4	72.2 ± 12.8		
SV_e_ (mL)	F	79.7 ± 14.7	76.9 ± 15.6	75.7 ± 14.9	77.4 ± 13.7	0.070	0.173
	M	84.6 ± 13.4	81.7 ± 16.6	83.6 ± 14.6	77.5 ± 14.4		
	All	82.0 ± 13.6	79.0 ± 15.4	79.3 ± 14.6	77.4 ± 13.3		
SV_Δ_ (mL)	F	−4.4 ± 1.5	−4.1 ± 1.2	−3.2 ± 3.1	−4.6 ± 2.4	0.668	0.097
	M	−5.3 ± 3.2	−5.2 ± 4.4	−8.0 ± 5.9	−6.1 ± 4.4		
	All	−4.8 ± 2.3	−4.6 ± 3.0	−5.4 ± 5.0	−5.3 ± 3.4		
*Q̇* (mL min^−^^1^)	F	4985 ± 1311	4949 ± 1329	5007 ± 1190	5047 ± 1138	0.454	0.271
	M	4274 ± 626	4274 ± 740	4436 ± 646	4135 ± 647		
	All	4662 ± 1074	4642 ± 1107	4748 ± 982	4633 ± 1021		
*Q̇*_i_ (mL min^−^^1^)	F	4845 ± 1266	4994 ± 1419	5047 ± 1288	5046 ± 1123	0.122	0.746
	M	4093 ± 605	4325 ± 782	4397 ± 647	4187 ± 705		
	All	4503 ± 1050	4690 ± 1172	4752 ± 1054	4656 ± 1015		
*Q̇*_e_ (mL min^−^^1^)	F	5134 ± 1359	4893 ± 1253	4975 ± 1095	5052 ± 1156	0.078	0.122
	M	4452 ± 701	4225 ± 728	4473 ± 779	4084 ± 636		
	All	4824 ± 1116	4589 ± 1058	4747 ± 995	4612 ± 1042		
*Q̇*_Δ_ (mL min^−^^1^)	F	−289 ± 129	101 ± 329	72 ± 260	−6 ± 95	0.001	0.642
	M	−359 ± 388	99 ± 306	−75 ± 621	103 ± 355		
	All	−321 ± 265^bd^	100 ± 303^a^	5 ± 441	44 ± 241^a^		

Data represent means ± SD (All *n* = 11, female *n* = 6, male *n* = 5). Significantly different from^a^S*f*_r_. ^b^R*f*_r_. ^c^6*f*_r_. ^d^D*f*_r_. *P *< 0.05. Abbreviations: 6F*f*_r_, 6 breaths min^−^^1^; Δ, inter‐breath phase difference (i minus e); D*f*_r_, optimization algorithm dynamic breathing frequency; e, mean expiration; F, female; i, mean inspiration; M, male; *Q̇*, cardiac output; R*f*_r_, RESPeRATE; S*f*_r_, spontaneous breathing; SV, stroke volume.

**TABLE A8:** Peak–valley differences for stroke volume (SV) and cardiac output ($\dot{Q}$).

		S*f*_r_	R*f*_r_	6F*f*_r_	D*f*_r_	Effect of condition *P*‐value	Sex × Condition *P*‐value
SV_Δi_ (mL)	F	5.3 ± 2.5	8.5 ± 3.8	9.5 ± 5.4	10.3 ± 6.2	0.006	0.895
	M	5.2 ± 1.0	9.8 ± 8.7	11.2 ± 6.1	10.1 ± 5.6		
	All	5.3 ± 1.8	9.1 ± 6.1	10.3 ± 5.5	10.2 ± 5.6		
SV_Δe_ (mL)	F	6.7 ± 3.0	9.9 ± 3.9	9.3 ± 1.1	11.1 ± 5.0	0.025	0.816
	M	5.7 ± 2.0	7.7 ± 4.3	8.0 ± 4.2	8.2 ± 5.5		
	All	6.2 ± 2.4	8.9 ± 4.0	8.7 ± 2.8	9.8 ± 5.2		
SV_ΔPV_ (mL)	F	−10.4 ± 3.3	−13.7 ± 3.3	−8.5 ± 12.4	−14.2 ± 4.5	0.384	0.248
	M	−10.5 ± 4.0	−14.9 ± 10.6	−17.9 ± 9.9	−14.9 ± 9.1		
	All	−10.5 ± 3.4	−14.2 ± 7.1	−12.8 ± 11.9	−14.5 ± 6.6		
SV_ΔPV_Ind_ (mL)	F	11.2 ± 2.7	11.1 ± 2.6	12.1 ± 3.3	13.0 ± 4.8	0.440	0.527
	M	14.8 ± 3.4	14.4 ± 8.2	17.2 ± 9.6	14.3 ± 7.0		
	All	12.8 ± 3.4	12.6 ± 5.8	14.4 ± 7.1	13.6 ± 5.6		
*Q̇*_Δi_ (mL min^−^^1^)	F	363.0 ± 301.2	878.2 ± 463.5	943.6 ± 474.3	1042.4 ± 694.5	<0.001	0.820
	M	304.2 ± 134.2	937.0 ± 760.7	1186.5 ± 764.9	1119.4 ± 734.7		
	All	340.6 ± 234.2^c^ *^d^*	904.9 ± 583.0	1054.0 ± 602.1^a^	1077.4 ± 677.3^a^		
*Q̇*_Δe_ (mL min^−^^1^)	F	517.8 ± 452.7	821.2 ± 485.3	860.1 ± 363.3	760.8 ± 449.2	< 0.001	0.209
	M	415.9 ± 113.7	686.7 ± 275.4	1020.5 ± 447.2	967.8 ± 535.2		
	All	473.6 ± 333.8^c^	760.0 ± 391.2	933.1 ± 391.2^a^	854.9 ± 476.6		
*Q̇*_ΔPV_ (mL min^−^^1^)	F	−751.2 ± 337.6	719.2 ± 1015.5	281.3 ± 1187.0	486.6 ± 1200.3	0.083	0.506
	M	−496.7 ± 754.3	−62.8 ± 1259.1	−105.1 ± 1727.6	27.1 ± 1508.6		
	All	−628.0 ± 571.1	363.8 ± 1147.4	105.7 ± 1392.5	277.8 ± 1299.4		
*Q̇*_ΔPV_Ind_ (mL min^−^^1^)	F	842.6 ± 344.6	1034.6 ± 560.9	1086.2 ± 474.4	941.9 ± 584.6	0.037	0.246
	M	1010.5 ± 196.8	1112.9 ± 514.1	1485.6 ± 699.5	1368.3 ± 746.6		
	All	918.1 ± 285.9	1070.2 ± 514.5	1267.7 ± 593.2	1135.7 ± 665.9		

Data represent means ± SD (All *n* = 11, female *n* = 6, male *n* = 5). Significantly different from ^a^S*f*_r_. ^b^R*f*_r_. ^c^6*f*_r_. ^d^D*f*_r_. *P *< 0.05.

Abbreviations: 6F*f*_r_, 6 breaths min^−^^1^; Δe, within‐expiration difference; Δi, within‐inspiration difference; ΔPV, inter‐breath phase peak–valley difference; ΔPV_Ind, breath‐phase‐independent peak–valley difference; D*f*_r_, optimization algorithm dynamic breathing frequency; F, female; M, male; *Q̇*, cardiac output; R*f*_r_, RESPeRATE; S*f*_r_, spontaneous breathing; SV, stroke volume.

**TABLE A9:** Mean values for total peripheral resistance (TPR) and pulse transit time (PTT).

		S*f*_r_	R*f*_r_	6F*f*_r_	D*f*_r_	Effect of condition *P*‐value	Sex × Condition *P*‐value
TPR (mmHg min L^−^^1^)	F	17.8 ± 3.9	17.9 ± 4.4	17.5 ± 3.1	17.3 ± 3.7	0.612	0.643
	M	21.2 ± 4.3	20.3 ± 5.2	19.7 ± 2.8	20.9 ± 4.1		
	All	19.3 ± 4.2	19.0 ± 4.7	18.5 ± 3.0	18.9 ± 4.1		
TPR_i_ (mmHg min L^−^^1^)	F	18.0 ± 4.0	17.7 ± 4.3	17.2 ± 3.0	17.1 ± 3.5	0.098	0.473
	M	21.9 ± 4.9	19.6 ± 5.2	19.2 ± 2.9	19.9 ± 4.1		
	All	19.8 ± 4.7	18.5 ± 4.6	18.1 ± 3.0	18.4 ± 3.9		
TPR_e_ (mmHg min L^−^^1^)	F	17.6 ± 3.9	18.3 ± 4.5	17.7 ± 3.2	17.6 ± 3.8	0.554	0.526
	M	20.4 ± 3.8	21.0 ± 5.4	20.2 ± 3.1	21.8 ± 4.2		
	All	18.9 ± 3.9	19.5 ± 4.9	18.8 ± 3.2	19.5 ± 4.4		
TPR_Δ_ (mmHg min L^−^^1^)	F	0.4 ± 0.7	−0.6 ± 0.8	−0.5 ± 1.1	−0.5 ± 0.6	<0.001	0.051
	M	1.5 ± 1.9	−1.4 ± 1.4	−0.9 ± 2.0	−1.9 ± 1.5		
	All	0.9 ± 1.4^bd^	−0.9 ± 1.2^a^	−0.7 ± 1.5	−1.1 ± 1.3^a^		
PTT (ms)	F	180 ± 23	183 ± 21	180 ± 21	183 ± 24	0.445	0.269
	M	210 ± 27	212 ± 24	215 ± 27	210 ± 22		
	All	193 ± 28	196 ± 26	196 ± 29	195 ± 26		
PTT_i_ (ms)	F	182 ± 24	185 ± 22	184 ± 23	183 ± 21	0.499	0.695
	M	213 ± 29	215 ± 23	215 ± 23	217 ± 24		
	All	196 ± 30	199 ± 26	198 ± 27	198 ± 27		
PTT_e_ (ms)	F	177 ± 22	180 ± 21	181 ± 24	177 ± 21	0.646	0.167
	M	207 ± 26	209 ± 24	205 ± 22	211 ± 29		
	All	191 ± 27	194 ± 26	192 ± 25	192 ± 30		
PTT_Δ_ (ms)	F	5.1 ± 2.7	4.7 ± 2.8	2.7 ± 6.8	6.4 ± 2.4	0.827	0.208
	M	6.4 ± 3.5	5.3 ± 2.5	10.4 ± 4.6	5.5 ± 8.0		
	All	5.7 ± 3.0	5.0 ± 2.6	6.2 ± 6.9	6.0 ± 5.4		

Data represent means ± SD (All *n* = 11, female *n* = 6, male *n* = 5). Significantly different from ^a^S*f*_r_. ^b^R*f*_r_. ^c^6*f*_r_. ^d^D*f*_r_. *P *< 0.05. Abbreviations: 6F*f*_r_, 6 breaths min^−^^1^; Δ, inter‐breath phase difference (i minus e); D*f*_r_, optimization algorithm dynamic breathing frequency; e, mean expiration; F, female; i, mean inspiration; M, male; PTT, pulse transit time; R*f*_r_, RESPeRATE; S*f*_r_, spontaneous breathing; TPR, total peripheral resistance.

**TABLE A10:** Peak–valley differences (± SD) for total peripheral resistance (TPR) and pulse transit time (PTT).

		S*f*_r_	R*f*_r_	6F*f*_r_	D*f*_r_	Effect of condition *P*‐value	Sex × Condition *P*‐value
TPR_Δi_ (mmHg min L^−^^1^)	F	1.4 ± 1.2	2.3 ± 1.0	2.3 ± 1.2	2.7 ± 1.8	0.001	0.176
	M	1.5 ± 0.6	3.1 ± 1.8	4.3 ± 2.2	3.9 ± 1.9		
	All	1.5 ± 1.0^c^	2.7 ± 1.4	3.2 ± 1.9^a^	3.2 ± 1.8		
TPR_Δe_ (mmHg min L^−^^1^)	F	1.9 ± 1.7	2.7 ± 1.5	2.7 ± 1.1	2.3 ± 1.4	0.004	0.058
	M	2.0 ± 0.6	3.2 ± 1.8	4.5 ± 2.4	4.7 ± 2.8		
	All	2.0 ± 1.3	3.0 ± 1.6	3.5 ± 1.9	3.4 ± 2.4		
TPR_ΔPV_ (mmHg min L^−^^1^)	F	−0.1 ± 2.8	−1.7 ± 3.5	−1.4 ± 3.4	−1.8 ± 3.7	0.037	0.284
	M	2.3 ± 3.6	−2.0 ± 4.6	−1.3 ± 6.4	−5.7 ± 2.9		
	All	1.0 ± 3.3*^d^*	−1.8 ± 3.9	−1.4 ± 4.7	−3.6 ± 3.7^a^		
TPR_ΔPV_Ind_ (mmHg min L^−^^1^)	F	3.1 ± 1.6	3.2 ± 1.5	3.5 ± 1.7	2.9 ± 1.8	0.190	0.180
	M	4.9 ± 1.1	4.7 ± 1.0	5.5 ± 2.1	5.6 ± 2.1		
	All	3.9 ± 1.6	3.9 ± 1.5	4.4 ± 2.1	4.2 ± 2.3		
PTT_Δi_ (ms)	F	11 ± 8	14 ± 4	17 ± 7	16 ± 8	<0.001	0.104
	M	9 ± 4	19 ± 10	22 ± 10	27 ± 17		
	All	10 ± 6^c^	16 ± 7	19 ± 9^a^	21 ± 13		
PTT_Δe_ (ms)	F	12 ± 8	15 ± 9	18 ± 7	16 ± 5	0.001	0.043
	M	10 ± 3	23 ± 11	28 ± 15	33 ± 23		
	All	12 ± 6^bc^	19 ± 10^a^	23 ± 12^a^	23 ± 17		
PTT_ΔPV_ (ms)	F	16 ± 10.0	9 ± 18	10 ± 25	21 ± 6	0.750	0.251
	M	16 ± 6	25 ± 10	34 ± 14	10 ± 45		
	All	16 ± 8	16 ± 17	21 ± 23	16 ± 29		
PTT_ΔPV_Ind_ (ms)	F	17 ± 9	16 ± 8	21 ± 8	17 ± 6	0.076	0.480
	M	24 ± 9	24 ± 7	32 ± 12	32 ± 18		
	All	20 ± 9	19 ± 8	26 ± 11	24 ± 14		

Data represent means ± SD (All *n* = 11, female *n* = 6, male *n* = 5). Significantly different from ^a^S*f*_r_. ^b^R*f*_r_. ^c^6*f*_r_. ^d^D*f*_r_. *P *< 0.05.

Abbreviations: 6F*f*_r_, 6 breaths min^−^^1^; Δe, within‐expiration difference; Δi, within‐inspiration difference; ΔPV, inter‐breath phase peak–valley difference; ΔPV_Ind, breath‐phase‐independent peak–valley difference; D*f*_r_, optimization algorithm dynamic breathing frequency; F, female; M, male; PTT, pulse transit time; R*f*_r_, RESPeRATE; S*f*_r_, spontaneous breathing; TPR, total peripheral resistance.

## APPENDIX B

### B.1

The following results (Tables B1–B5) present the mean, mean inspiration and mean expiration data for all variables. Correlation coefficients for blood pressure variables are also presented in full for all breathing conditions.

**TABLE B1:** Mean values for blood pressure variables (mmHg).

	S*f*_r_	R*f*_r_	6F*f*_r_	D*f*_r_	Effect of condition *P*‐value
SBP (mmHg)	120.0 ± 12.8	117.2 ± 11.2	117.1 ± 12.1	115.5 ± 9.9	0.358
SBP_i_ (mmHg)	117.7 ± 12.8	114.7 ± 11.6	114.0 ± 12.1	112.0 ± 11.0	0.157
SBP_e_ (mmHg)	122.3 ± 12.8	119.7 ± 11.5	120.1 ± 12.8	119.1 ± 9.4	0.659
SBP_Δ_ (mmHg)	−4.6 ± 2.3	−5.0 ± 5.6	−6.1 ± 6.0	−7.0 ± 4.7	0.158
DBP (mmHg)	71.4 ± 8.8	68.9 ± 7.7	70.7 ± 9.3	69.9 ± 9.1	0.412
DBP_i_ (mmHg)	70.8 ± 8.8	68.3 ± 8.6	69.8 ± 9.9	68.6 ± 9.5	0.326
DBP_e_ (mmHg)	72.1 ± 8.9	69.5 ± 7.2	71.7 ± 9.0	71.2 ± 8.9	0.482
DBP_Δ_ (mmHg)	−1.2 ± 1.2	−1.2 ± 3.4	−1.9 ± 3.0	−2.5 ± 2.5	0.288

Data represent means ± SD (*n* = 11). Abbreviations: 6F*f*_r_, 6 breaths min^−^^1^; DBP, diastolic blood pressure; D*f*_r_, optimization algorithm dynamic breathing frequency; e, mean expiration; i, mean inspiration; R*f*_r_, RESPeRATE; SBP, systolic blood pressure; S*f*_r_, spontaneous breathing; Δ, inter‐breath phase difference (i minus e; mmHg).

**TABLE B2:** Correlation coefficients between SBP_Δi_ and SBP, SBP_Δe_ and SBP and the DBP equivalents.

Correlation	S*f*_r_	R*f*_r_	6F*f*_r_	D*f*_r_
SBP/SBP_Δi_	0.97	0.83	0.87	0.95
SBP/SBP_Δe_	0.97	0.87	0.89	0.88
DBP/DBP_Δi_	0.98	0.68	0.55	0.79
DBP/DBP_Δe_	0.99	0.78	0.81	0.83

Data represent Pearson's product moment correlation coefficient (*n* = 11). Abbreviations: 6F*f*_r_, 6 breaths min^−^^1^; DBP, diastolic blood pressure (mmHg); D*f*_r_, optimization algorithm dynamic breathing frequency; R*f*_r_, RESPeRATE; SBP, systolic blood pressure (mmHg); S*f*_r_, spontaneous breathing; Δe, within‐expiration difference; Δi, within‐inspiration difference.

**TABLE B3:** Mean values for heart rate (*f*_c_).

	S*f*_r_	R*f*_r_	6F*f*_r_	D*f*_r_	Effect of condition *P*‐value
*f*_c_ (beats min^−^^1^)	58.6 ± 8.5^cd^	60.0 ± 8.5	62.4 ± 9.0^a^	62.3 ± 9.4^a^	<0.001
*f*_c,i_ (beats min^−^^1^)	58.4 ± 8.2^bcd^	62.9 ± 8.8^a^	64.4 ± 9.3^a^	64.7 ± 9.2^a^	<0.001
*f*_c,e_ (beats min^−^^1^)	58.8 ± 9.0	58.3 ± 8.5	60.5 ± 9.5	59.8 ± 9.8	0.125
*f*_c,Δ_ (beats min^−^^1^)	−0.5 ± 2.6^bd^	4.6 ± 3.2^a^	3.8 ± 5.2	4.9 ± 3.3^a^	0.005

Data represent means ± SD (*n* = 11). Significantly different from ^a^S*f*_r_. ^b^R*f*_r_. ^c^6*f*_r_. ^d^D*f*_r_. *P *< 0.05. Abbreviations: 6F*f*_r_, 6 breaths min^−^^1^; Δ, inter‐breath phase difference (i minus e); D*f*_r_, optimization algorithm dynamic breathing frequency; e, mean expiration; *f*_c_, heart rate; i, mean inspiration; R*f*_r_, RESPeRATE; S*f*_r_, spontaneous breathing.

**TABLE B4:** Mean values for stroke volume (SV) and cardiac output ($\dot{Q}$).

	S*f*_r_	R*f*_r_	6F*f*_r_	D*f*_r_	Effect of condition *P*‐value
SV (mL)	79.6 ± 13.8	76.7 ± 14.8	76.6 ± 13.3	74.8 ± 12.9	0.027
SV_i_ (mL)	77.1 ± 14.0	74.4 ± 14.4	73.9 ± 12.4	72.2 ± 12.8	0.013
SV_e_ (mL)	82.0 ± 13.6	79.0 ± 15.4	79.3 ± 14.6	77.4 ± 13.3	0.070
SV_Δ_ (mL)	−4.8 ± 2.3	−4.6 ± 3.0	−5.4 ± 5.0	−5.3 ± 3.4	0.668
$\dot{Q}$ (mL min^−^^1^)	4662 ± 1074	4642 ± 1107	4748 ± 982	4633 ± 1021	0.454
$\dot{Q}$_i_ (mL min^−^^1^)	4503 ± 1050	4690 ± 1172	4752 ± 1054	4656 ± 1015	0.122
$\dot{Q}$_e_ (mL min^−^^1^)	4824 ± 1116	4589 ± 1058	4747 ± 995	4612 ± 1042	0.078
$\dot{Q}$_Δ_ (mL min^−^^1^)	−321 ± 265^bd^	100 ± 303^a^	5 ± 441	44 ± 241^a^	0.001

Data represent means ± SD (*n* = 11) Significantly different from ^a^S*f*_r_. ^b^R*f*_r_. ^c^6*f*_r_. ^d^D*f*_r_. *P *< 0.05. Abbreviations: 6F*f*_r_, 6 breaths min^−^^1^; Δ, inter‐breath phase difference (i minus e); D*f*_r_, optimization algorithm dynamic breathing frequency; e, mean expiration; i, mean inspiration; *Q̇*, cardiac output; R*f*_r_, RESPeRATE; S*f*_r_, spontaneous breathing; SV, stroke volume.

**TABLE B5:** Mean values for total peripheral resistance (TPR) and pulse transit time (PTT).

	S*f*_r_	R*f*_r_	6F*f*_r_	D*f*_r_	Effect of condition *P*‐value
TPR (mmHg min L^−^^1^)	19.3 ± 4.2	19.0 ± 4.7	18.5 ± 3.0	18.9 ± 4.1	0.612
TPR_i_ (mmHg min L^−^^1^)	19.8 ± 4.7	18.5 ± 4.6	18.1 ± 3.0	18.4 ± 3.9	0.098
TPR_e_ (mmHg min L^−^^1^)	18.9 ± 3.9	19.5 ± 4.9	18.8 ± 3.2	19.5 ± 4.4	0.554
TPR_Δ_ (mmHg min L^−^^1^)	0.9 ± 1.4^bd^	−0.9 ± 1.2^a^	−0.7 ± 1.5	−1.1 ± 1.3^a^	<0.001
PTT (ms)	193 ± 28	196 ± 26	196 ± 29	195 ± 26	0.445
PTT_i_ (ms)	196 ± 30	199 ± 26	198 ± 27	198 ± 27	0.499
PTT_e_ (ms)	191 ± 27	194 ± 26	192 ± 25	192 ± 30	0.646
PTT_Δ_ (ms)	5.7 ± 3.0	5.0 ± 2.6	6.2 ± 6.9	6.0 ± 5.4	0.827

Data represent means ± SD (*n* = 11). Significantly different from ^a^S*f*_r_. ^b^R*f*_r_. ^c^6*f*_r_. ^d^D*f*_r_. *P *< 0.05. Abbreviations: 6F*f*_r_, 6 breaths min^−^^1^; Δ, inter‐breath phase difference (i minus e); D*f*_r_, optimization algorithm dynamic breathing frequency; e, mean expiration; i, mean inspiration; PTT, pulse transit time; R*f*_r_, RESPeRATE; S*f*_r_, spontaneous breathing; TPR, total peripheral resistance.
